# Supplementary material for: Identification of photoperiod-induced specific miRNAs in the adrenal glands of Sunite sheep (Ovis aries)
Source: Front Vet Sci. 2022 Jul 22;9:888207. doi: 10.3389/fvets.2022.888207 (PMC9354845; doi:10.3389/fvets.2022.888207)
Supplement: Supplementary file 1 [file Data_Sheet_1.ZIP › Supplementary file/miRDeep2 file.zip!/miRDeep2 file/Novel_Structure/SP42A2/Novel_648.pdf]

Provisional ID : NC_019473.2_30663
Score total : 2.8
Score for star read(s) : -1.3
Score for read counts : 0
Score for mfe : 2.5
Score for randfold : 1.6
Score for cons. seed :
Total read count : 33
Mature read count : 33
Loop read count : 0
Star read count : 0

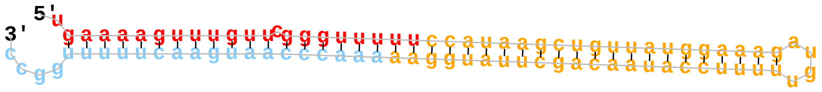

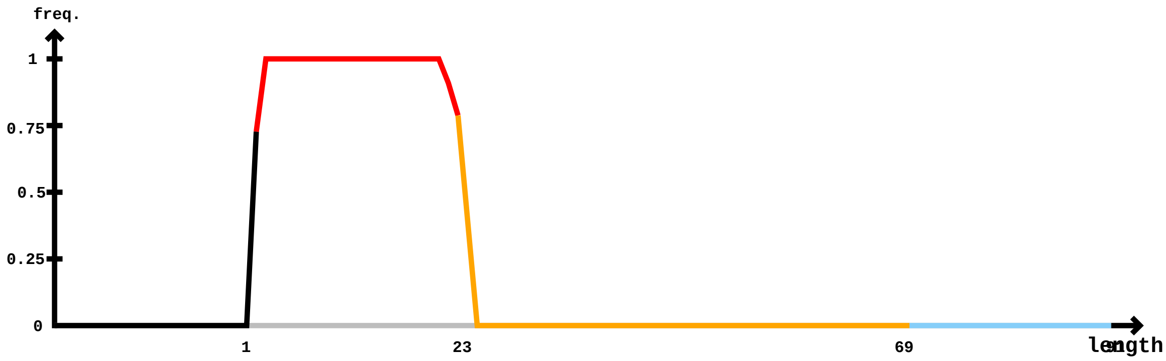

Mature **Star**

```
5'- cccacagacacuggguuggugaaaaguuuguucggguuuuuccauaagcuguuauggaaagauguuuuuccauaacagcuuauggaaaaacccaaugaacuuuuuggccaa  -3'   exp
    ((((.......))))(((((.(((((((((((.((((((((((((((((((((((((((((....)))))))))))))))))))))))))))))))))))))))..)))))    reads   mm   sample
    ...................ugaaaaguuuguucggguuu........................................................................    3       0    seq
    ...................ugaaaaguuuguucggguuuu.......................................................................    4       0    seq
    ...................ugaaaaguuuguucggguuuuu......................................................................    16      0    seq
    ...................ugaaaaguuuguucggguuuuuc.....................................................................    1       0    seq
    ....................gaaaaguuuguucggguuuuu......................................................................    8       0    seq
    ....................gaaaaguuuguucggguuuuuc.....................................................................    1       0    seq
```
